# Die Entwicklung der psychischen Gesundheit bei hochaltrigen Individuen während der COVID-19-Pandemie und die Rolle sozialer Unterstützung

**DOI:** 10.1007/s00103-023-03660-0

**Published:** 2023-01-30

**Authors:** Sina K. Gerhards, Alexander Pabst, Steffi G. Riedel-Heller, Melanie Luppa

**Affiliations:** Institut für Sozialmedizin, Arbeitsmedizin und Public Health (ISAP), Medizinische Fakultät, Universität Leipzig, Philipp-Rosenthal-Str. 55, 04103 Leipzig, Deutschland

**Keywords:** Coronavirus-Pandemie, Depressivität, Ängstlichkeit, Psychosoziale Gesundheit, Hohes Lebensalter, Coronavirus-Pandemic, Depressiveness, Anxiety, Psychosocial health, Late Life

## Abstract

**Hintergrund:**

Die Bevölkerungsgruppe der Hochaltrigen gehört zu den Hochrisikogruppen in Bezug auf einen schweren Erkrankungsverlauf und erhöhte Mortalität bei Ansteckung mit dem Coronavirus SARS-CoV-2 (engl.: Severe Acute Raspiratory Syndrom Coronavirus 2). Sie ist durch die COVID-19-Pandemie selbst, aber auch durch Maßnahmen des Gesundheitsschutzes möglicherweise einem höheren Risiko für psychische Belastung ausgesetzt. Es soll untersucht werden, wie sich Symptomatiken von Depressivität, Ängstlichkeit und Somatisierung im Verlauf der Pandemie verändern und welche Rolle die soziale Unterstützung dabei spielt.

**Methoden:**

Mittels zweier schriftlicher Befragungen von *n* = 156 Hochaltrigen in den Zeiträumen Mai bis Juni 2020 sowie März bis Mai 2021 wurden neben soziodemografischen Daten Faktoren der psychischen Belastung (Depressivität, Ängstlichkeit, Somatisierung) sowie die wahrgenommene soziale Unterstützung erfasst. Das mittlere Alter der Befragten betrug 87,20 Jahre (*SD* = 4,65; Altersspanne = 77,68–96,75 Jahre; 2020) bzw. 88,03 Jahre (*SD* = 4,63; Altersspanne = 78,52–97,62; 2021). Die Daten wurden mittels Wilcoxon-t-Tests und generalisierter linearer Regressionsmodelle analysiert.

**Ergebnisse:**

Es lässt sich eine signifikante Zunahme der Ausprägung psychischer Belastung hinsichtlich Depressivität, Ängstlichkeit und Somatisierung erkennen. Höhere Werte der psychischen Belastung im Jahr 2020 sind mit einer höheren psychischen Belastung im Jahr 2021 assoziiert. Eine stärkere wahrgenommene soziale Unterstützung im Jahr 2020 ist mit geringerer Depressivität ein Jahr später assoziiert.

**Schlussfolgerung:**

Bei hochaltrigen Menschen ist im Verlauf der COVID-19-Pandemie bis Mai 2021 eine Zunahme der psychischen Belastung zu verzeichnen. Sie sollten durch präventive Angebote unterstützt werden, um einer weiteren Zunahme der Symptomatik vorzubeugen. Der Ausbau von sozialer Unterstützung könnte v. a. bei der Prävention depressiver Symptomatik sinnvoll sein.

## Abstract (en)

**Background:**

The elderly population is one of the high-risk groups with regard to a severe course of disease and increased mortality when infected with the coronavirus SARS-CoV‑2 (Severe Acute Raspiratory Syndrom Coronavirus 2). This group may be at higher risk for psychological strains from the COVID-19 pandemic itself but also from the health protection measures. The aim is to examine how symptoms of depressiveness, anxiety, and somatization change over the course of the pandemic and which role social support plays in that.

**Methods:**

Using two written surveys of *n* = 156 elderly participants in the periods May to June 2020 and March to May 2021, sociodemographic data, factors of psychological strain (depressiveness, anxiety, and somatization), as well as the perceived social support were recorded. The mean age of the respondents was 87.20 years (*SD* = 4.65; age range = 77.68–96.75 years) and 88.03 years (*SD* = 4.63; age range = 78.52–97.62 years) for 2020 and 2021, respectively. Data were analyzed using Wilcoxon t‑tests and generalized linear regression models.

**Results:**

A significant increase in the expression of psychological strain with regards to depressiveness, anxiety, and somatization can be identified. Higher scores of psychological strains in 2020 are associated with a higher psychological strain in 2021. Higher perceived social support in 2020 is associated with lower depressiveness one year later.

**Conclusion:**

An increase in psychological strain has been observed in the elderly population over the course of the COVID-19 pandemic until May 2021. This population should be supported by preventive programs to avert a further increase in symptoms. The expansion of social support could be useful, especially in the prevention of depressive symptoms.

## Einleitung

Im November 2019 wurden erste Fälle einer Infektion mit dem Virus SARS-CoV‑2 (engl.: severe respiratory syndrom coronavirus 2) bekannt und am 11.03.2020 erklärte die Weltgesundheitsorganisation (WHO) COVID-19 (Corona Virus Disease 19) zu einer Pandemie [1]. Menschen ab 60 Jahren und insbesondere Menschen ab 80 Jahren wurden als Hochrisikogruppe für einen schweren oder sogar tödlichen Verlauf identifiziert, wobei das Risiko bei Männern noch höher ist als bei Frauen [2, 3]. Es wurden Maßnahmen zur Eindämmung der Pandemie eingeführt. Dazu zählten Abstands- und Hygieneregelungen, die Maskenpflicht und Quarantänemaßnahmen sowie nationale Ausgangssperren und Empfehlungen für Besuchsbeschränkungen und -verbote in Altenpflegeheimen und Krankenhäusern, um insbesondere ältere Menschen und Personen mit Vorerkrankungen vor einer Ansteckung zu schützen [4–6]. Am 22.03.2020 trat der erste Corona-Lockdown in Kraft, mit einer Beschränkung der Kontakte auf bis zu 2 Personen. Außerdem wurden Geschäfte geschlossen und es durften keine religiösen Zusammentreffen stattfinden [5]. Nach den Lockerungen der Maßnahmen während des Sommerplateaus der Infektionszahlen wurde im Dezember 2020 der Einzelhandel abermals geschlossen und es gab Empfehlungen zu erneuten Kontaktbeschränkungen [7]. Ebenfalls im Dezember begannen die ersten Impfungen gegen SARS-CoV‑2, wobei ältere Menschen priorisiert wurden [8]. Von Januar bis Mai 2021 folgte der zweite Lockdown mit verschärften Kontaktbeschränkungen. Private Zusammenkünfte waren nur innerhalb des eigenen Haushaltes und mit maximal einer weiteren Person erlaubt. Gottesdienste waren unter strengen Hygiene- und Abstandsregelungen erlaubt [9]. Insgesamt wirkte sich die COVID-19-Pandemie stark auf das soziale Zusammenleben aus, wobei ältere Menschen als besonders schutzbedürftig identifiziert wurden [10].

Soziale Eingebundenheit ist essentiell für die körperliche und psychische Gesundheit. Auch unabhängig von dem Pandemiegeschehen und damit assoziierten staatlichen Maßnahmen ist bekannt, dass soziale Isolation und damit verbundene Gefühle der Einsamkeit in der Bevölkerungsgruppe der Älteren mit einem erhöhten Risiko für kardiovaskuläre und psychische Erkrankungen verbunden sind [11–13]. Ein größeres Ausmaß an sozialer Unterstützung hingegen ist assoziiert mit einer geringeren psychischen Belastung wie depressiven Symptomen [14, 15]. Eine systematische Übersicht von Wang et al. [16] zeigte, dass weniger soziale Unterstützung mit einer höheren psychischen Belastung assoziiert ist, insbesondere mit erhöhtem Schweregrad depressiver Symptomatik. In einer Studie von Levkovich et al. zeigte sich eine negative Assoziation zwischen sozialer Unterstützung und depressiver Symptomatik während der Pandemie bei Menschen im Alter von 65 bis 95 Jahren in Israel [17]. Außerdem gibt es Hinweise auf einen Zusammenhang zwischen geringer sozialer Unterstützung mit vermehrten Angstsymptomen [16].

Während der Pandemie wurden die Rahmenbedingungen im Hinblick auf soziale Eingebundenheit und Unterstützung problematischer, da die Abstands- und Quarantäneregelungen die Lage in Bezug auf die soziale Isolation älterer und hochaltriger Menschen zusätzlich erschwerten. In einer repräsentativen Querschnittsbefragung von Menschen im hohen Alter (ab 80 Jahren) zur Wahrnehmung der COVID-19-Pandemie in Deutschland beschrieb die Mehrheit (54,3 %) negative Auswirkung der Pandemie auf ihre sozialen Kontakte [18]. Auf der anderen Seite gaben 90,9 % der Befragten an, mind. 2 Bezugspersonen zu haben, und 40 % verbrachten häufig Zeit mit Verwandten, Freunden oder Bekannten [18].

Aufgrund der Zunahme von Belastung, z. B. durch Ausgangsbeschränkungen, Sorge vor Ansteckung und Abstandsregelungen im Rahmen der Pandemie, wurden negative Auswirkungen auf die mentale Gesundheit befürchtet [19]. Zum jetzigen Zeitpunkt gibt es jedoch nur wenige Studien, die sich mit den psychischen Auswirkungen der Pandemie auf die Bevölkerungsgruppe der Hochaltrigen beschäftigen, was dazu führt, dass Menschen im Alter von 80 Jahren und älter im Vergleich zu anderen Bevölkerungsgruppen nur unzureichend abgebildet sind.

Die bisherige Forschung zeigt ein heterogenes Bild in Bezug auf die psychische Belastung älterer Menschen während der COVID-19-Pandemie [20]. Es gibt Studien, die eine Abnahme der subjektiven Lebenszufriedenheit und eine Zunahme psychischer Belastung in Form von depressiven Symptomen bei älteren Menschen berichten [21]. Parlapani et al. weisen in dem von ihnen verfassten Review darauf hin, dass die Studienergebnisse auf einen protektiven Faktor des Alters in Bezug auf psychische Belastung während der COVID-19-Pandemie hinweisen, es aber Unterschiede in den verschiedenen älteren Teilpopulationen, beispielsweise derer mit hohem Maß an sozialer Isolation oder körperlicher Multimorbidität, geben könnte [22]. Weitere Studien weisen auf eine hohe Resilienz der älteren und hochaltrigen Bevölkerung hin und beschreiben ein stabiles Niveau psychischer Gesundheitsfaktoren [23–25], wobei Tilburg et al. [25] gleichzeitig auf einen Anstieg der Einsamkeit bei den untersuchten 65- bis 102-jährigen Personen hinweisen und Kivi et al. [24] zudem eine Zunahme des negativen Affekts (Gefühls- und Gemütslebens) bei Menschen im Alter von 65–71 Jahren beschreiben.

Während junge Menschen zu Beginn der Pandemie deutliche psychische Belastungen zeigten, erschienen alte Menschen resilienter [19]. Dabei beziehen sich Studien zur psychischen Gesundheit in der älteren Bevölkerung meist auf einen Untersuchungszeitraum zu Beginn der Pandemie und der Anteil von hochaltrigen Menschen im Alter von 80 Jahren und älter ist meist unterrepräsentiert oder nicht inkludiert. Es ist bisher wenig darüber bekannt, inwieweit sich die psychische Belastung in dieser Gruppe im weiteren Verlauf der Pandemie entwickelte. In der vorliegenden Untersuchung widmen wir uns daher folgenden Fragestellungen:

1. Wie verändert sich die psychische Belastung im Sinne von Depressivität, Ängstlichkeit und Somatisierung im Verlauf der COVID-19-Pandemie?
2. Welche Rolle spielt die wahrgenommene soziale Unterstützung im ersten Erhebungszeitraum bei der Vorhersage der psychischen Belastungsfaktoren Depressivität, Ängstlichkeit und Somatisierung ein Jahr später?

## Methoden

### Studiendesign und Stichprobe

Bei der vorliegenden Studie handelt es sich um eine Befragungsstudie mit längsschnittlichem Design. Im Zeitraum von Mai bis Juni 2020, kurz nach dem ersten Lockdown, wurden Fragebögen zum Thema der psychischen Bewältigung der Pandemie postalisch an *n* = 378 Personen im hohen Alter in Leipzig und naher Umgebung versendet, von denen *n* = 197 (52,1 %) einen ausgefüllten Fragebogen zurücksendeten. An der zweiten Erhebung von März bis Mai 2021, zum Ende des zweiten Lockdowns, nahmen *n* = 156 (79,9 %) Personen teil.

Die kontaktierten Personen stammten aus einem Pool von ehemaligen Studienteilnehmer/-innen, die ihr Einverständnis gegeben hatten, für weitere Studien kontaktiert zu werden.

### Erhebungsverfahren

Soziodemografische Faktoren wurden mittels standardisierter Fragen erfasst und beinhalteten das Alter (in Jahren), das Geschlecht (männlich/weiblich), das Bildungsniveau (kategorisiert in Anlehnung an die „Comparative Analysis of Social Mobility in Industry Nations“ – CASMIN: niedrig, mittel, hoch) und den Familienstand (verheiratet/mit Partner, geschieden/ledig, verwitwet).

Die wahrgenommene soziale Unterstützung wurde mit Hilfe des ENRICHD Social Support Inventory (ESSI; [26]) erhoben. Dieses umfasst 5 Items, die jeweils auf einer 5‑Punkt-Likert-Skala („niemals“ bis „immer“, 1–5) bewertet werden können. Höhere Mittelwertscores stehen für eine größere wahrgenommene soziale Unterstützung. Es handelt sich um eine etablierte Skala mit guten psychometrischen Eigenschaften [26, 27].

Das Brief Symptom Inventory 18 (BSI-18; [28]) erfasst die psychische Belastung auf den 3 Subskalen „Depressivität“, „Ängstlichkeit“ und „Somatisierung“. Die Skalen bestehen aus jeweils 6 Items, die auf einer 5‑Punkt-Likert-Skala bewertet werden können („überhaupt nicht“ bis „sehr stark“, 0–4). Das BSI-18 weist gute psychometrische Eigenschaften auf [28].

### Statistische Analysen

Die Daten wurden mittels der Programme SPSS Statistics 27.0 (Statistical Package for Social Science Inc., IBM Corp., Armonk, NY, USA [29]) und Stata 16.0 SE (Stata Statistical Software, Stata Corp., College Station, TX, USA [30]) analysiert. Neben der deskriptiven Statistik wurden für die Ermittlung von Mittelwertunterschieden nichtparametrische Wilcoxon-Tests durchgeführt. Aufgrund einer schiefen Verteilung der Outcome-Variablen konnten keine klassischen linearen Modelle angewendet werden. Daher wurden generalisierte lineare Regressionsmodelle mit einer Gamma-Verteilung und Log-Link-Funktion [31] zur Ermittlung der Assoziationen der Outcome-Variablen Depression, Angst und Somatisierung im Jahr 2021 (t2) mit soziodemografischen Daten und sozialer Unterstützung im Jahr 2020 (t1) durchgeführt und robuste Standardfehler berechnet. Die eingeschlossenen Variablen weisen ein metrisches Skalenniveau auf, mit Ausnahme von Geschlecht (männlich/weiblich), Bildung (CASMIN; niedrig/mittel/hoch) und Familienstand (verheiratet/ledig, geschieden/verwitwet). Geschlechtsunterschiede in Bezug auf die Ausprägung psychischer Belastung (Depression, Angst, Somatisierung) wurden aufgrund der nichtparametrischen Verteilung mittels Mann-Whitney-U-Test analysiert. Des Weiteren wurden Non-Responder-Analysen bezüglich des Alters und der Faktoren psychischer Belastung durchgeführt. Hier zeigt sich, dass Personen, die an der zweiten Befragung nicht mehr teilnahmen (*n* = 41) höhere Werte psychischer Belastung zum Baseline-Messzeitpunkt aufwiesen (Depression: *M* = 2,91, *SD* = 3,44; Angst: *M* = 2,74, *SD* = 2,33; Somatisierung: *M* = 4,91, *SD* = 2,97) und etwas älter waren (*M* = 90,49, *SD* = 4,94) als die Teilnehmer/-innen, die an beiden Studien teilnahmen (Depression: *M* = 0,40, *SD* = 1,08; Angst: *M* = 0,31, *SD* = 0,81; Somatisierung: *M* = 1,47, *SD* = 2,42; Alter: *M* = 88,03, *SD* = 4,63).

## Ergebnisse

Die Stichprobe der vorliegenden Befragung besteht aus *n* = 156 Personen höheren Alters mit einem mittleren Alter von 87,20 Jahren (SD = 4,65; Altersspanne = 77,68–96,75 Jahre) zum Zeitpunkt der ersten Erhebung von Mai bis Juni 2020 und einem mittleren Alter von 88,03 Jahren (SD = 4,63; Alterspanne = 78,52–97,62 Jahre) zum Zeitpunkt der Folgeerhebung von März bis Mai 2021. 56,4 % der Studienteilnehmer/-innen waren weiblich. Die große Mehrheit (99,36 %) wohnte im eigenen Haushalt, lediglich eine Person (0,64 %) im Seniorenheim. Soziodemografische Charakteristika der Stichprobe sind in Tab. 1 zusammengefasst.

**Table 1:** 

	Baseline (2020)	Follow-up (2021)
*Alter; M (Range; SD)*	87,20 (77,68–96,75; 4,65)	88,03 (78,52–97,62; 4,63)
*Geschlecht; n (%)*		
Weiblich	88 (56,4)	88 (56,4)
Männlich	68 (43,6)	68 (43,6)
*Familienstand*^*a*^*; n (%)*		
Verheiratet	68 (43,6)	66 (42,6)
Ledig/geschieden	15 (9,6)	14 (9,0)
Verwitwet	73 (46,8)	75 (48,4)
*Bildungsstand (CASMIN); n (%)*		
Niedrig	52 (34,2)	52 (34,2)
Mittel	40 (26,3)	40 (26,3)
Hoch	60 (39,5)	60 (39,5)

^a^Fehlende Werte bei Familienstand 2021: *n* = 1 (0,64 %)

Im Verlauf der COVID-19-Pandemie bis Mai 2021 haben Depressivität, Ängstlichkeit und Somatisierung zugenommen (Tab. 2). Zwischen 2020 und 2021 zeigt sich ein signifikanter Anstieg der Depressivität (0,40 vs. 2,12; *z* = −7,29; *p* < 0,001) mit einer hohen Effektstärke von *r* = 0,61, wobei 56,3 % der Befragten im Jahr 2021 einen höheren Mittelwert auf der entsprechenden Skala aufwiesen. Bei 41,7 % blieb der mittlere Score depressiver Symptomatik konstant und bei 2,1 % verringerte er sich. Ebenfalls zeigt sich eine signifikante Zunahme der Ängstlichkeit (0,31 vs. 2,23; *z* = 8,40; *p* < 0,001) mit einer hohen Effektstärke von *r* = 0,70. Hier erhöhte sich der Mittelwert der Ängstlichkeitsskala bei 66,0 % der Teilnehmer/-innen. Bei 33,3 % blieb der Wert konstant und er verringerte sich bei 0,7 %. Auch die Somatisierungssymptome nahmen signifikant zu (1,47 vs. 4,74; *z* = −9,26; *p* < 0,001) mit einer hohen Effektstärke von *r* = 0,80. Hier erhöhte sich der Mittelwert der Somatisierungsskala bei 85,0 % der Befragten. Bei 12,0 % blieb der Wert konstant und bei 3,0 % verringerte er sich. Die wahrgenommene soziale Unterstützung änderte sich zwischen den beiden Erhebungszeiträumen nicht signifikant.

**Table 2:** 

	2020^a^ M (SD)	2021^b^ M (SD)	*z*	*p*	Effektstärke (*r*)
Depressivität^c^	0,40 (1,08)	2,12 (2,81)	−7,29	**<0,001**	0,61
Ängstlichkeit^d^	0,31 (0,81)	2,23 (2,38)	−8,40	**<0,001**	0,70
Somatisierung^e^	1,47 (2,42)	4,74 (3,87)	−9,26	**<0,001**	0,80
Soziale Unterstützung^f^	4,41 (0,76)	4,47 (0,68)	−1,68	0,092	–

Für die Berechnung der deskriptiven Statistik wurden die Summenscores der BSI-Skalen verwendet

*M* Mittelwert, *SD* Standardabweichung, *z* z-Wert, *p* p-Wert

^a^Mai bis Juni 2020; fehlende Werte: Unterstützung staatl. Maßnahmen: *n* = 2 (1,3 %), Depressivität: *n* = 7 (4,5 %), Ängstlichkeit *n* = 5 (3,2 %), Somatisierung: *n* = 10 (6,4 %)

^b^März bis Mai 2021; fehlende Werte: Depressivität, Ängstlichkeit: *n* = 11 (7,1 %), Somatisierung: *n* = 12 (7,7 %)

^c^Subskala Depression des Brief Symptom Inventory (BSI-18)

^d^Subskala Angst des Brief Symptom Inventory (BSI-18)

^e^Subskala Somatisierung des Brief Symptom Inventory (BSI-18)

^f^ENRICHD Social Support Inventory (ESSI)

Mit Ausnahme eines Geschlechtsunterschiedes bei der depressiven Symptomatik im Erhebungszeitraum März bis Mai 2021, wobei Frauen im Vergleich zu Männern eine stärkere depressive Symptomausprägung aufwiesen (2021: *U* = 2246,00; *z* = −1,985; *p* = 0,047), gab es keine Geschlechtsunterschiede in Bezug auf die Ausprägung der Depressivität (2020: *U* = 2405,00; *z* = −1,736; *p* = 0,083), Ängstlichkeit (2020: *U* = 2683,00; *z* = −0,771; *p* = 0,441/2021: *U* = 2287,00; z = 1,209; *p* = 0,227) oder Somatisierungssymptome (2020: *U* = 2580,00; *z* = −0,187 *p* = 0,851/2021: *U* = 2475,50; *z* = −0,214; *p* = 0,830).

Tab. 3 zeigt die Assoziationen der soziodemografischen Faktoren und sozialer Unterstützung im Jahr 2020 mit den Zielparametern zur psychischen Gesundheit im Jahr 2021 in der Bevölkerungsgruppe der Hochaltrigen. Die soziale Unterstützung zeigt unabhängig von den soziodemografischen Charakteristika und der Ausprägung depressiver Symptomatik zur ersten Erhebung 2020 einen signifikanten Zusammenhang mit der Ausprägung der Depressivität im Jahr 2021, wobei höhere Werte wahrgenommener sozialer Unterstützung im Jahr 2020 mit einer geringeren Depressivität im Jahr 2021 assoziiert sind (*exp* *(b)* = 0,619; *z* = −2,90; *p* = 0,004).

**Table 3:** 

	Psychische Belastung 2021								
	Depressivität^a^			Ängstlichkeit^b^			Somatisierung^c^		
	*Exp* *(b)*	*95* *%-KI*		*Exp* *(b)*	*95* *%-KI*		*Exp* *(b)*	*95* *%-KI*	
*Jeweiliger psych. Gesundheits-Parameter im Jahr 2020*	7,300**	*1,770*	*30,120*	5,233***	2,060	13,230	3,916***	2,417	6,344
*Alter*	1,023	0,964	1,085	1,017	0,972	1,064	1,010	0,980	1,041
*Geschlecht*									
Weiblich	*Ref.*			*Ref.*			*Ref.*		
Männlich	0,940	0,542	1,630	0,769	0,482	1,225	0,891	0,645	1,231
*Bildung*	*x*^2^(2) = 0,68			*x*^2^(2) = 0,60			*x*^2^(2) = 1,98		
Niedrig	*Ref.*			*Ref.*			*Ref.*		
Mittel	0,831	0,480	1,438	1,228	0,723	2,086	1,027	0,732	1,440
Hoch	1,029	0,556	1,905	1,131	0,714	1,791	1,244	0,894	1,731
*Familienstand*	*x*^2^(2) = 2,88			*x*^2^(2) = 1,41			*x*^2^(2) = 3,42		
Verheiratet	*Ref.*			*Ref.*			*Ref.*		
Ledig/geschieden	2,118	0,880	5,097	1,457	0,757	2,803	0,645	0,398	1,045
Verwitwet	1,133	0,777	2,281	1,027	0,635	1,660	0,972	0,468	1,337
*Soziale Unterstützung*	0,619**	0,448	0,856	0,901	0,637	1,275	1,025	0,812	1,779

Generalisiertes lineares Modell mit Gamma-Verteilung und Log-Link-Funktion. Für alle kategorialen Variablen wurden Omnibustests durchgeführt. Für die Analyse wurden Mittelwert-Scores der jeweiligen BSI-Skala verwendet

*Exp(b)* exponentiated coefficient, *KI* Konfidenzintervall, *Ref.* Referenzkategorie

**p < 0,010, ***p < 0,005

^a^Brief Symptom Inventory 18 (BSI-18) Subskala Depressivität, *n* = 136

^b^Brief Symptom Inventory 18 (BSI-18) Subskala Ängstlichkeit, *n* = 133

^c^Brief Symptom Inventory 18 (BSI-18) Subskala Somatisierung, *n* = 128

Erwartungsgemäß zeigt sich, dass die jeweilige Ausprägung psychischer Belastung im Jahr 2020 mit der Ausprägung der psychischen Belastung im Jahr 2021 assoziiert ist, wobei verstärkte depressive Symptome im Jahr 2020 mit höheren Depressivitäts-Scores im Jahr 2021 assoziiert sind (*exp* *(b)* *=* 7,300; *z* = 2,75; *p* = 0,006). Höhere Ängstlichkeitswerte im Jahr 2020 sind mit mehr Ängstlichkeit im Jahr 2021 (*exp* *(b)* *=* 5,233; *z* = 3,48; *p* = 0,001) und höhere Somatisierungswerte 2020 mit verstärkten Somatisierungssymptomen 2021 (*exp* *(b)* *=* 3,916; *z* = 5,54; *p* < 0,001) assoziiert.

## Diskussion

Ein Ziel der vorliegenden Analyse war die Untersuchung der Veränderung der Symptomausprägung von Depressivität, Ängstlichkeit und Somatisierung zu Beginn der COVID-19-Pandemie (Mai bis Juni 2020) und etwa ein Jahr später (März bis Mai 2021) bei hochaltrigen Menschen.

Die hochaltrige Bevölkerung zeigte sich zum Zeitpunkt der ersten Erhebungswelle im Jahr 2020 nur sehr gering belastet und widerstandsfähig gegenüber einer psychischen Belastung durch die Pandemie. Die Werte zum zweiten Erhebungszeitpunkt im Jahr 2021 waren demgegenüber signifikant erhöht.

Petrowski et al. [32] erhoben vor der COVID-19-Pandemie die Werte psychischer Belastung mittels BSI-18 in der deutschen Allgemeinbevölkerung. Die Altersgruppe 80–95 Jahre (*n* = 119) wies Depressivität mit einem Mittelwert von 2,43 (*SD* = 3,84), Ängstlichkeit mit einem Mittelwert von 1,82 (*SD* = 3,14) und Symptome der Somatisierung mit einem Mittelwert von 3,89 (*SD* = 3,75) auf. Im Vergleich dazu liegen in der vorliegenden Befragung im Jahr 2021 die Mittelwerte der untersuchten 79- bis 98-Jährigen bei der Depressivität bei 2,12 (*SD* = 2,81), bei Ängstlichkeit bei 2,23 (*SD* = 2,38) und bei der Somatisierung bei 4,74 (*SD* = 3,87). Aus Ermangelung der Konfidenzintervalle der Mittelwerte in der zitierten Studie von Petrowski et al. [32] lässt sich an dieser Stelle zwar keine eindeutige statistische Bewertung vornehmen. Es deutet sich allerdings an, dass die Werte der hochaltrigen Individuen im Jahr 2021 in Bezug auf die Depressivität und Somatisierungssymptomatik leicht unter den vorpandemischen Vergleichswerten liegen könnten, wohingegen die Ausprägung der Ängstlichkeit etwas über den vorpandemischen Vergleichswerten liegen könnte.

Insgesamt zeigt sich eine signifikante Zunahme aller 3 Faktoren psychischer Belastung zwischen den beiden Erhebungszeiträumen. Das bedeutet genauer, dass sich Depressivität, Ängstlichkeit und Somatisierungssymptome im Vergleich zu denen im Jahr 2020 verschlechterten. Hochaltrige Individuen, die zu Beginn der Pandemie höhere Werte psychischer Belastung im Sinne von Depressivität, Ängstlichkeit und Somatisierung zeigten, wiesen wahrscheinlicher auch ein Jahr später höhere Depressivität‑, Ängstlichkeits- und Somatisierungswerte auf. Personen im hohen Alter, die psychisch belastet sind, sollten also im Fokus von Interventionen zur Reduktion psychischer Belastungssymptomatik stehen. Es ist bedeutsam, spezifische und an die Bedürfnisse der hochaltrigen Menschen angepasste Interventionen zu etablieren.

Der Anstieg psychischer Belastung bei den befragten hochaltrigen Individuen ist eindrücklich und weitere Untersuchungen sollten erfolgen, um diese Untersuchungen mit größeren Stichproben durchzuführen und den weiteren Verlauf der Symptomatik zu analysieren.

In der vorliegenden Studie wurde auch der Einfluss der wahrgenommenen sozialen Unterstützung im Jahr 2020 auf Depressivität, Ängstlichkeit und Somatisierungssymptome im Jahr 2021 in der Bevölkerungsgruppe der hochaltrigen Menschen in Deutschland untersucht. Eine größere wahrgenommene soziale Unterstützung ist mit einer geringeren Depressivität ein Jahr später assoziiert. Dies steht in Einklang mit vorpandemischen Forschungsergebnissen (z. B. [14, 15, 33]), die berichten, dass eine stärkere wahrgenommene soziale Unterstützung mit weniger depressiven Symptomen assoziiert ist. Auch steht das Ergebnis in Übereinstimmung mit der systematischen Übersichtsarbeit von Wang et al. [16], die einen Zusammenhang von geringer wahrgenommener sozialer Unterstützung mit einer stärkeren depressiven Symptomausprägung zeigen, und den Ergebnissen von Levkovich et al. [17], die einen Zusammenhang von starker wahrgenommener sozialer Unterstützung und weniger depressiven Symptomen berichteten. Entgegen der Studie von Wang et al. [8] zeigt sich der Zusammenhang von geringer sozialer Unterstützung mit stärkeren Ausprägungen der Ängstlichkeit in der vorliegenden Stichprobe hochaltriger Menschen nicht. Es scheint andere wichtige prädiktive Faktoren für das Ausmaß der Ängstlichkeit und Somatisierung zu geben, die es in weiteren Studien zu untersuchen gilt.

Auch zu Zeiten der COVID-19-Pandemie scheint die soziale Unterstützung in der hochaltrigen Bevölkerung ein bedeutsamer Faktor für die Vorhersage von Depressivität zu sein und einen vielversprechenden Ansatz für entsprechende Präventionsprogramme zu bieten. Weitere Untersuchungen mit größeren Stichproben bezüglich der Zusammenhänge zwischen sozialer Unterstützung mit Depressivität und auch Ängstlichkeit und Somatisierung sind notwendig, um ein noch besseres Verständnis zu generieren und spezifische, zielgruppengerechte Interventionsmöglichkeiten ableiten zu können.

### Limitationen

Neben den Stärken der vorliegenden Studie, wie der Befragung einer schwer erreichbaren und bisher kaum untersuchten Stichprobe hochaltriger Menschen während der COVID-19-Pandemie, sowie dem längsschnittlichen Design können folgende Limitationen angeführt werden. Es handelt sich um eine eher kleine regionale Stichprobe, was die Aussagekraft der Ergebnisse möglicherweise einschränkt. Außerdem beziehen sich die Ergebnisse auf Personen, die in Privathaushalten leben. Somit kann keine Aussage über die psychische Belastung von Menschen gemacht werden, die z. B. im Altenpflegeheim leben. Diese waren aber möglicherweise von der Pandemie und den assoziierten Besuchs- und Kontaktverboten in besonderer Weise betroffen. Zudem zeigen Non-Responder-Analysen, dass hochaltrige Individuen, die zum ersten Erhebungszeitpunkt bereits eine höhere psychische Belastung aufwiesen, an der zweiten Erhebung häufiger nicht teilnahmen. Dies könnte bedeuten, dass die vorliegenden Ergebnisse das reale Bild in der hochaltrigen Bevölkerung unterschätzen und die Belastung im zweiten Erhebungszeitraum höher gewesen sein könnte. Zukünftige Studien müssten die Entwicklung der psychischen Belastungsindikatoren während des weiteren Pandemieverlaufes ab Mai 2021 untersuchen.

## Schlussfolgerungen

Die vorliegende Arbeit deutet darauf hin, dass hochaltrige Individuen während der COVID-19-Pandemie der akuten Belastung mit psychischer Widerstandsfähigkeit begegnet sind. Chronische, längerfristige Belastungen durch die Pandemie scheinen allerdings nachteilige Effekte auf die psychische Gesundheit hochaltriger Menschen zu haben. Auch wenn sich hochaltrige Menschen zu Beginn der Pandemie nur wenig psychisch belastet zeigten, ist im Verlauf der Pandemie bis Mai 2021 eine Zunahme psychischer Belastung im Sinne von Depressivität, Ängstlichkeit und Somatisierungssymptomatik sichtbar. Es erscheint daher ratsam, Personen mit ausgeprägteren Angst‑, Depressions- und/oder Somatisierungssymptomen durch supportive und präventive Maßnahmen zu unterstützen, um einer weiteren Zunahme der psychischen Symptomatik entgegenzuwirken. Maßnahmen und Interventionen zur sozialen Unterstützung, inklusive der Förderung der sozialen Einbindung, sind für die Prävention von Depressivität bei hochaltrigen Menschen relevant.
